# Relationship between Vessel Formation and Seasonal Changes in Leaf Area of Evergreen and Deciduous Species with Different Vessel Arrangements

**DOI:** 10.3390/plants10010100

**Published:** 2021-01-06

**Authors:** Sayaka Takahashi, Erina Takahashi

**Affiliations:** 1 Faculty of Life and Environmental Sciences, Shimane University, Nishikawatsu-cho, Matsue-shi, Shimane 690-8504, Japan; erina@life.shimane-u.ac.jp; 2 Graduate School of Agriculture, Kyoto University, Oiwake-cho Kitashirakawa, Sakyo-ku, Kyoto 606-8502, Japan; 3 Field Science Education and Research Center, Kyoto University, Oiwake-cho Kitashirakawa, Sakyo-ku, Kyoto 606-8502, Japan

**Keywords:** diffuse-porous tree, leaf appearance, quantitative leaf phenology, Richards’ growth function, radial-porous tree, ring-porous tree, semi-ring porous trees, time course of leaf area, vessel arrangement, vessel porosity

## Abstract

To discuss the diversity of morphological traits and life strategies of trees, the functional relationship between leaf expansion and vessel formation must be clarified. We compared the temporal relationship among tree species with different leaf habits and vessel arrangements. Twigs, leaves, and trunk core samples were periodically acquired from 35 sample trees of nine species in a temperate forest in Japan. We quantitatively estimated leaf expansion using a nonlinear regression model and observed thin sections of twigs and trunks with a light microscope. Almost all of the first-formed vessels in twigs, which formed adjacent to the annual ring border, were lignified with a leaf area between 0% and 70% of the maximum in all species. The first-formed vessels in trunks lignified between 0% and 95% of the maximum leaf area in ring-porous deciduous *Quercus serrata* and ring-(radial-)porous evergreen *Castanopsis cuspidate*. Their lignification occurred earlier than in diffuse-porous deciduous *Liquidambar styraciflua*, diffuse-porous evergreen *Cinnamomum camphora* and *Symplocos prunifolia*, and radial-porous evergreen *Quercus glauca* and *Quercus myrsinifolia*. The timing varied in semi-ring-porous deciduous *Acanthopanax sciadophylloides* and diffuse-porous evergreen *Ilex pedunculosa*. The observed differences in the timing of vessel formation after leaf appearance were reflected in their differing vessel porosities and were connected to the different life strategies among tree species.

## 1. Introduction

Trees growing in temperate forests have various wood structures and leaf habits. Evergreen and deciduous broad-leaved trees grow in the same or overlapping areas and have different vessel arrangements that can be characterized as ring-porous, diffuse-porous, semi-ring-porous [1], and radial-porous [2,3]. Ring-porous trees have wider vessels in the wood, which are formed in spring, then narrower vessels are formed after the summer. Diffuse-porous trees have vessels with a similar diameter across the annual ring. Semi-ring-porous trees are intermediate in characteristics between ring-porous trees and diffuse-porous trees, and radial-porous trees are characterized by vessels in a radial direction. Differences in the size and distribution of vessels lead to seasonal variations in organ development based on their relationship with water conduction efficiency.

These morphological varieties influence tree growing habitats and their life strategies. One of the most important factors for tree growth is the timing of leaf expansion and the beginning of water conducting tissue formation. Leaves and water conducting tissues are closely related via transpiration and water transport. It has been reported that relationships exist between leaf area and the conducting tissue [4,5], and the temporal relationships between leaf appearance and the formation of the first-formed vessels, which are formed adjacent to the annual ring border [6,7,8,9]. Studies of deciduous species have shown that the timing of the first-formed vessel formation in trunks relative to leaf appearance in ring-porous trees was earlier than in diffuse-porous trees [6,7,10]. This different timing of vessel formation in early spring is highly controlled by growth hormones [11,12]. The cambium in ring-porous trees is more sensitive to indole-3-acetic acid stimulation than in diffuse-porous trees [13]. Previous studies have shown that the first-formed vessel formation from the bud bases to the trunk base begins simultaneously with bud break in ring-porous trees, whereas the vessels form gradually from the bud bases to the trunk base in diffuse-porous trees [7,10]. The time lag of the first-formed vessel formation between twigs and trunks shows the same tendency as between the vessel formation of twigs and leaf appearance [7,10]. The twig vessel formation begins within ±2 weeks of leaf appearance in ring-porous and diffuse-porous deciduous trees [7]. However, the timing of vessel formation in twigs and stems after leaf appearance is still unclear.

The differences in vessel width are closely related to functional differences. The wide vessels of ring-porous tree trunks form around the time of leaf appearance [6,7,14,15,16,17] until full leaf expansion [7,18], and during leaf and shoot expansion [9]. A trade-off relationship exists for the wide vessels whereby they are able to contribute considerably to water transport in the current year [19] but are susceptible to freezing in the winter [19,20]. By contrast, relatively small vessels of diffuse-porous tree trunks mature from 2 weeks to several weeks after leaf appearance [6,7,21] and after full leaf expansion [7,18]. The vessels of diffuse-porous trees can recover from freezing during winter [19,22,23], though they have low water conduction efficiency [19]. Furthermore, ring-porous trees form leaves and vessels synchronously to meet water transport requirements, whereas diffuse-porous trees show asynchronous tissue formation patterns [24]. Unlike diffuse-porous trees, the vessel diameter of ring-porous trees changes with leaf formation [24]. Takahashi et al. [24] published a quantitative analysis of the relationship between vessel formation and leaf expansion after leaf appearance, though it is not sufficient.

A previous study has shown that different porosities may not affect the timing of vessel formation in trunks relative to leaf appearance in semi-ring-porous deciduous trees and in evergreen trees with various types of porosities [25]. The time lag between the first-formed vessel lignification of trunks and leaf appearance in ring-porous evergreen trees is not always short. Furthermore, this time lag is not always long in diffuse-porous evergreen trees [25]. Similar to ring-porous and diffuse-porous deciduous trees, the first-formed vessel lignification of twigs is concurrent with leaf appearance in evergreen trees, irrespective of different vessel arrangements [25]. Nevertheless, the vessel formation pattern between leaf appearance and full leaf expansion in evergreen species and semi-ring-porous deciduous species remains unclear.

These studies are limited to the qualitative observation of leaf expansion, though quantitative observation is important for understanding the connections between the morphological traits of vessel and leaf formation and their functional traits. The quantitative formation processes of leaves and vessels must be examined to elucidate the relationships between leaf expansion and vessel formation with regard to physiological functions such as photosynthesis and water transport. Studies have qualitatively examined leaf phenology (e.g., leaf appearance, full leaf expansion, and leaf fall), as quantitative examination has proven difficult [24]. The transpiration capacity of broad-leaved trees increases with increasing leaf area, and leaf area reaches the maximum at the same time as the maximum of transpiration capacity [26,27] and photosynthetic capacity [27,28,29]. Increases in the potential capacities of transpiration and photosynthesis in correspondence with leaf expansion can be estimated by quantitative observation of the leaf area. There are few relevant studies, and a quantitative examination of the leaf area time courses in relation to vessel formation [18], as well as the time courses of leaf area, have not been modeled. The timing of vessel formation in twigs and trunks in relation to the time of full leaf expansion, when leaf area reaches the maximum, in deciduous and evergreen species is still not clear.

To clarify the relationship between leaf expansion and vessel formation, we examined the timing of the first-formed vessel lignification of twigs and trunks in connection with the leaf area time course. We compared their temporal relationship among tree species with different leaf habits and vessel arrangements. The time course of leaf area was quantified by fitting a nonlinear regression model [30] in order to examine leaf area growth with regard to physiological functions. Clarifying the effects of morphological traits on the temporal relationship of leaf phenology and vessel formation and on their life strategies can provide clues about the diversity of trees species growing in temperate forests.

## 2. Results

### 2.1. Relationship between Leaf Area and Twig Vessel Formation

**Figure 1:**
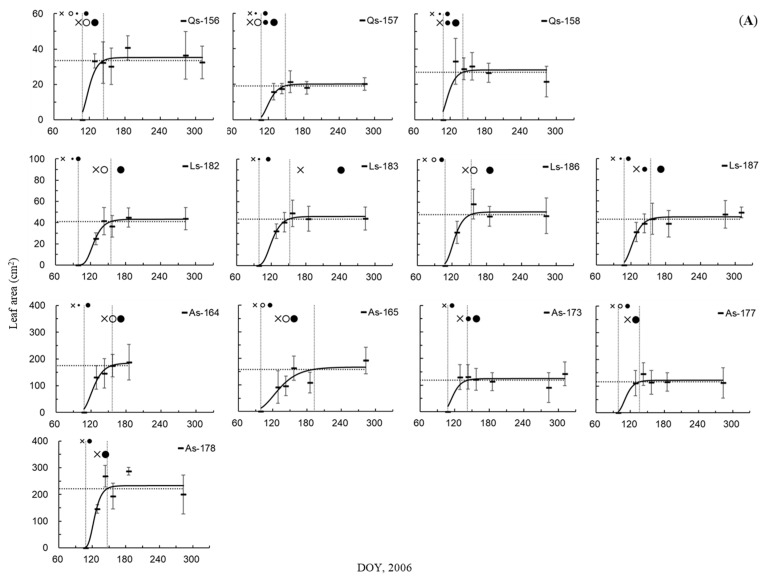

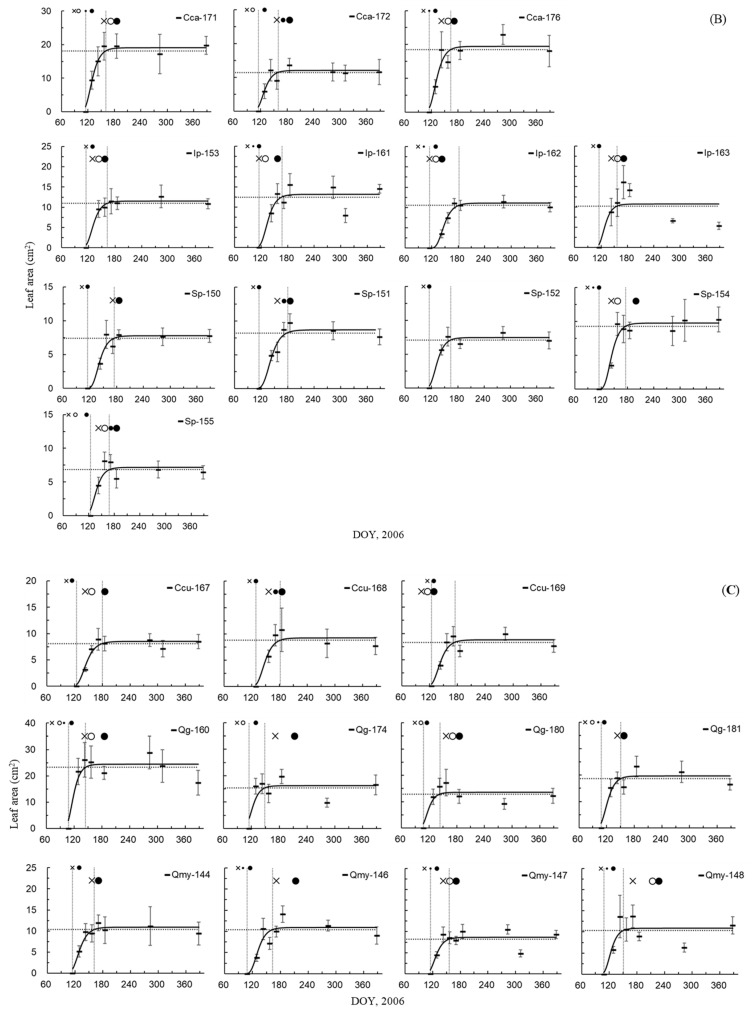
Nonlinear regression model of leaf area time course and day of year (DOY) of the first-formed vessel lignification in twigs (small symbols) and trunks (large symbols). Crosses—no vessel; open circles—vessel enlargement; small, filled circles—at least one of the first-formed vessels lignified; large, filled circles—almost all of the first-formed vessels lignified; vertical dotted line—95% of the maximum leaf area; horizontal dotted line—DOY of 0% and 95% of the maximum leaf area; hyphens—means; bars—standard deviations. (**A**) Deciduous ring-porous *Quercus serrata*, diffuse-porous *Liquidambar styraciflua*, and semi-ring-porous *Acanthopanax sciadophylloides*; (**B**) evergreen diffuse-porous *Cinnamomum camphora*, *Ilex pedunculosa*, and *Symplocos prunifolia*; (**C**) evergreen ring-(radial-)porous *Castanopsis cuspidate*, and radial-porous *Quercus glauca* and *Quercus myrsinifolia*.

**Figure 2:**
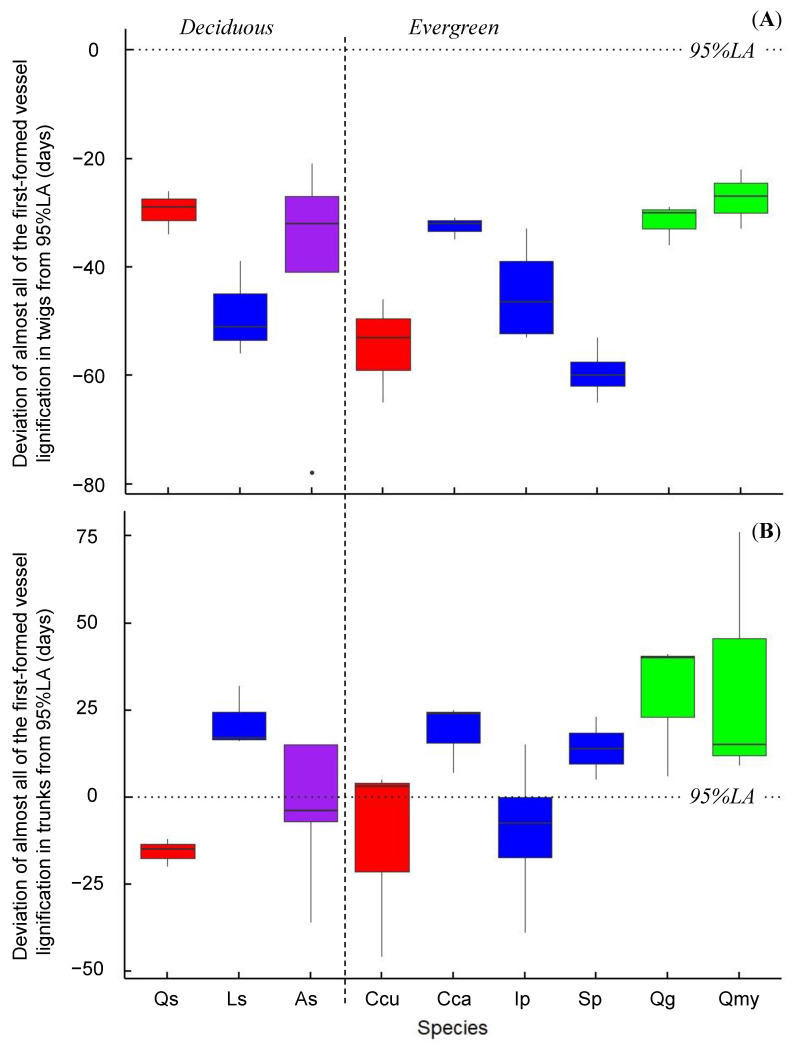
Deviation in lignification of almost all of the first-formed vessels in (**A**) twigs and (**B**) trunks from full leaf expansion (95% of the maximum leaf area; 95%LA). Dotted lines—95%LA; a dashed line—a boundary between deciduous and evergreen species; the shadowed area of boxes—between 25th and 75th percentile; **red**—ring-porous; **blue**—diffuse-porous; **purple**—semi-ring-porous; **green**—radial-porous; Qs—*Quercus serrata*; Ls—*Liquidambar styraciflua*; As—*Acanthopanax sciadophylloides*; Ccu—*Castanopsis cuspidata*; Cca—*Cinnamomum camphora*; Ip—*Ilex pedunculosa*; Sp—*Symplocos prunifolia*; Qg—*Quercus glauca*; Qmy—*Quercus myrsinifolia.*

As shown in Figure 1 and Figure 2A, almost all of the first-formed vessels in twigs were lignified when the leaf area was between 2% and 70% of the maximum leaf area (2%LA and 70%LA, respectively) in the deciduous trees ring-porous *Quercus serrata* (28–44%) and semi-ring-porous *Acanthopanax sciadophylloides* (12–59%), and in the evergreen trees diffuse-porous *Cinnamomum camphora* (43–50%) and *Ilex pedunculosa* (2–43%), and radial-porous *Q. glauca* (28–70%) and *Q. myrsinifolia* (5–66%). Vessel enlargement or lignification of at least one of the first-formed vessels was observed before the period corresponding to 0%LA in some sample trees of these species. Almost all of the first-formed vessels were lignified during the period between 1%LA and 31%LA in some sample trees of diffuse-porous deciduous *Liquidambar styraciflua* (22, 31%) and ring-(radial-)porous evergreen *Castanopsis cuspidata* (3, 14%), and diffuse-porous evergreen *Symplocos prunifolia* (1, 5%), whereas almost all of the first-formed vessels were lignified before the period corresponding to 0%LA in the other trees of these species.

### 2.2. Relationship between Leaf Area and Trunk Vessel Formation

In ring-porous deciduous *Q. serrata* (Qs) and ring-(radial-)porous evergreen *C. cuspidate* (Ccu), almost all of the first-formed vessels in trunks were lignified during the period between 0%LA and 95%LA (Figure 1A,C and Figure 2B); 79%, 71%, and 84% of LA in Qs-156, -157, and -158, respectively; 95%LA in Ccu-167, -168; and 14%LA in Ccu-169. In diffuse-porous deciduous *L. styraciflua* and the evergreen trees diffuse-porous *C. camphora* and *S. prunifolia*, and radial-porous *Q. glauca* and *Q. myrsinifolia*, almost all of the first-formed vessels were lignified after 95%LA (Figure 1 and Figure 2B). In semi-ring-porous deciduous *A. sciadophylloides* (As) and diffuse-porous evergreen *I. pedunculosa* (Ip), almost all of the first-formed vessels were lignified during the period between 0%LA and 95%LA in some sample trees (80%, 90%, and 93%LA in As-165, -177, and -178, respectively, and 93%, 89%, and 36%LA in Ip-153, -161, and -162, respectively). Almost all of the first-formed vessels were lignified after 95%LA in the three other trees, in which vessel enlargement or lignification of at least one of the first-formed vessels was observed before 95%LA (Figure 1A,B and Figure 2B). Current-year vessels in the trunk of *S. prunifolia* (Sp-152) were not observed through the sampling period nor in additional sampling performed on 10 October and 7 November (Figure 1B).

## 3. Discussion

In previous research of deciduous species, the timing of the first-formed vessel formation in twigs relative to leaf phenology did not differ between ring- and diffuse-porous trees [10]. Twig vessel elements began to lignify prior to bud opening [14], and cell division at the bud bases began before or at the same time as bud break in both ring- and diffuse-porous trees [10]. Previous studies have shown that lignification of almost all of the first-formed vessels occurs within ±2 weeks of leaf appearance [7], e.g., 0 week and 0–3 weeks before leaf appearance in ring-porous *Quercus serrata* and diffuse-porous *Liquidambar styraciflua*, respectively [25]. In the present study, almost all of the first-formed vessels in twigs lignified during the period when leaf area was between 0% and 70% of the maximum, not only in deciduous species but also in evergreen species (Figure 1 and Figure 2A), though evergreen species have perennial leaves. This suggests that the first-formed vessels of twigs are complete at the time of leaf expansion in all species. These vessels are estimated to be used for effective water transport when transpiration and photosynthesis reach their maxima. Twig vessel enlargement and lignification of at least one of the first-formed vessels occurred before the period corresponding to 0%LA in some sample trees (Figure 1), showing that twig vessel formation would begin before leaf appearance.

Previous studies of deciduous species have shown that the timing of first-formed vessel formation in trunks relative to leaf phenology differs between ring- and diffuse-porous trees [6,7,10]. Studies on ring-porous trees have shown that the beginning of cambial activity [31] and enlargement [32] of trunk vessels coincide with the unfolding of the first leaves. The completion of secondary wall deposition in trunks began at −1 to 3 weeks after leaf expansion [6] or contemporaneous with bud burst [15], and within ±1 week of leaf budding [18]. The first-formed vessel lignification of trunks occurred after bud break [33], contemporaneous with leaf appearance [14], leaf expansion [16], and at −2 to 4 weeks after leaf appearance until full leaf expansion [7]. The perforation of end walls, which is estimated to coincide with the beginning of water transport, was also observed after leaf budding until leaf expansion [34,35]. By contrast, diffuse-porous trees were found to complete secondary wall deposition of the first-formed vessels in trunks 4–9 weeks after leaf expansion [6] and lignification 2–8 weeks after leaf appearance and after full leaf expansion [7]. The first-formed vessel formation in trunks relative to leaf appearance in ring-porous trees tends to occur earlier than that in diffuse-porous trees [6,7,10]. The results of the present study are similar to these previous findings. Lignification of the first-formed trunk vessels occurred until full leaf expansion (71–84%LA) in ring-porous *Q. serrata* and after full leaf expansion in diffuse-porous *L. styraciflua* (Figure 1A and Figure 2B), i.e., 2–4 weeks before and more than 7 weeks after leaf appearance, respectively [25]. Previous studies have reported that the wide vessels of the inner ring in ring-porous species are not used for water transport [23,36,37,38,39], and sap flow increases with the increase in leaf area index (LAI) [40]. It has been suggested that ring-porous species may need to form the first-formed vessels from twigs to trunks for full leaf expansion, when the water requirement for photosynthesis and transpiration reaches the maximum. By contrast, the vessels of several outer rings in diffuse-porous species may be used for water transport [22,23,36,37]. The water conductivity gradually decreases from the outermost xylem to inner sapwood [41,42]. It has been suggested that diffuse-porous species may form the first-formed vessels of trunks for water transport after full leaf expansion in the first year and for transpiration of leaves in the following several years.

In their study of evergreen species, ring-(radial-)porous trees have wider vessels of pore zone than those of non-pore zone. Hirano [43] reported that secondary wall deposition of the first-formed vessels in trunks is completed concurrently with leaf appearance in ring-(radial-)porous *Castanopsis sieboldii*. It was reported that the first-formed vessels of trunks lignified not only before but also long after leaf appearance in ring-(radial-)porous *C. cuspidata* [25]. In the present study, almost all of the first-formed vessels were lignified before full leaf expansion (14–95%LA; Figure 1C and Figure 2B), the same as for ring-porous deciduous species. It is estimated that ring-porous evergreen species may appear to complete the development of the first-formed vessels from twigs to trunks prior to full leaf expansion and achieve effective water transport for transpiration of current year leaves. Nevertheless, in *C. cuspidata*, vessels in several rings adjacent to the cambial zone retain their water transport ability for several years [44], and tyloses were found in old sapwood rings of *C. sieboldii* [45]. This indicates that the vessels in several outer rings may contribute to water transport in ring-(radial-)porous evergreen species. This is different from ring-porous deciduous species, suggesting that evergreen species require functional vessels all year round to transport water for transpiration and photosynthesis of perennial leaves. The mechanism for refilling the wide vessels with water after winter embolism is still unclear, and the functional phenophase of leaf/vessel formation in relation to the water transport mechanism is a subject for future study.

Radial-porous wood has a similar vessel porosity to diffuse-porous wood due to the change in vessel size in radial directions. Lignification of almost all of the first-formed vessels in trunks occurred after full leaf expansion in radial-porous evergreen *Q. glauca* and *Q. myrsinifolia* (Figure 1C and Figure 2B), as observed in diffuse-porous deciduous species. However, Hirano [43] reported that secondary wall deposition of the first-formed vessels in trunks was completed at the time of leaf appearance in radial-porous evergreen *Lithocarpus edulis*. Vessel density of radial-porous wood is uneven in the tangential direction and the trunk shape of radial-porous species sometimes has irregularities, suggesting that the timing of the beginning of vessel formation is different given the tangential position along the trunk.

A previous study of diffuse-porous evergreen species showed that almost all of the first-formed vessels in trunks are lignified after leaf appearance [25]. We found that the timing of almost all of the first-formed vessels’ lignification occurs after full leaf expansion in diffuse-porous evergreen species, whereas this timing occurs both before (36–93%LA) and after full leaf expansion in *I. pedunculosa* (Figure 1B and Figure 2B). Diffuse-porous evergreen species have perennial leaves and functional vessels [44] that can begin to participate in photosynthesis and water transport even before the appearance of new leaves [46]. This suggests that most of these species do not need to produce leaves and trunk vessels within a short time period.

In a previous study, lignification of almost all of the first-formed vessels in trunks of some semi-ring-porous deciduous *A. sciadophylloides* individuals occurred concurrently with leaf appearance, though this occurred after in other *A. sciadophylloides* individuals [25]. The timing was both before and after full leaf expansion in the present study (Figure 1A and Figure 2B). *A. sciadophylloides* had narrower first-formed vessels than those in the ring-porous species, and sometimes the porosity was similar to that of diffuse-porous species. Saitoh et al. [45] reported that tyloses of *A. sciadophylloides* were present in old sapwood rings. However, Umebayashi et al. [23] categorized *A. sciadophylloides* as a ring-porous species, and its wide vessels were used for water transport primarily during the year in which they are formed, similar to other ring-porous species. Thus, *A. sciadophylloides* appears to have intraspecific variations in the phenophase of leaf/vessel formation, vessel arrangement, and their functions.

The time lag between the first-formed vessels of twigs and trunks demonstrated the same tendency as the time lag between twig vessel formation and leaf appearance in all species of the present study (Figure 1 and Figure 2), which is the same as the results of deciduous species in the previous study [7,10]. The first-formed vessel lignification of twigs at 0.5–1.5 cm from the bud base occurred within a short period of leaf appearance. This suggests that the length of the time lag between leaf appearance and first-formed vessel formation of trunks depends on the spreading speed of vessel formation from the bud bases to the trunk.

## 4. Materials and Methods 

### 4.1. Study Site and Sampled Trees

The study site was a secondary forest of deciduous and evergreen trees of the Kamigamo Experimental Forest Station (35°04′ N, 135°46′ E, 109–225 m above sea level), Kyoto University, Kyoto Prefecture, Japan. The mean annual temperature over the 30 year period of 1976–2005 was 14.7 °C, the highest average temperature was 31.8 °C in August, and the lowest average temperature was −0.9 °C in January. The mean annual precipitation was 1523 mm. Environmental data were obtained from the Forest Research Station of Graduate School of Agriculture, Kyoto University [47].

**Figure 3:**
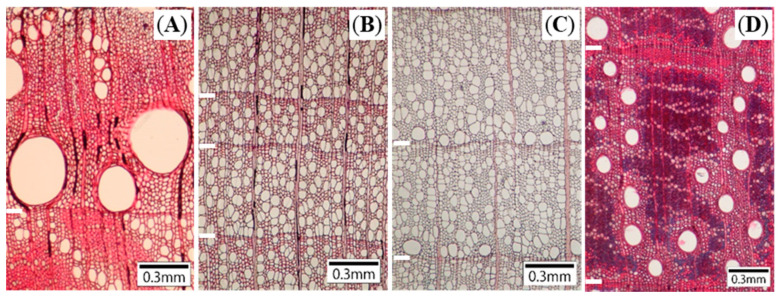
Light micrographs of cross-sections in (**A**) ring-porous *Quercus serrata*, (**B**) diffuse-porous *Liquidambar styraciflua,* (**C**) semi-ring-porous *Acanthopanax sciadophylloides*, and (**D**) radial-porous *Q. myrsinifolia*. White bars—ring borders.

Sample trees from nine species with four types of vessel porosity were selected (Table 1; Figure 3): ring-porous deciduous *Quercus serrata*; diffuse-porous deciduous *Liquidambar styraciflua*; semi-ring-porous deciduous *Acanthopanax sciadophylloides*; ring-(radial-)porous evergreen *Castanopsis cuspidata*; diffuse-porous evergreen *Cinnamomum camphora*, *Ilex pedunculosa*, and *Symplocos prunifolia*; and radial-porous evergreen *Q. glauca* and *Q. myrsinifolia*. Study trees were selected from individuals with relatively straight trunks, a diameter at breast height (1.3 ± 0.3 m above the ground) in the range 12–56 cm, and a total height of 7–29 m (Table 1). All *L. styraciflua* and *Q. myrsinifolia* were planted trees.

### 4.2. Sampling

Cylindrical wood core samples and twigs with leaves were collected from 3–5 trees per species (Table 1). Twigs and leaves that were located in the higher part of crown were cut using 12 m long pruners or by climbing with 3 m long pruners. Twigs that had grown in the previous year were regarded as one-year-old twigs. Vessel formation was examined in one-year-old twigs at 0.5–1.5 cm from the bud base, with the exception of the occasional 2–4 year-old twigs in *Acanthopanax sciadophylloides*. Cylindrical wood core samples were collected at breast height using a Mattson increment borer (7 mm diameter; Haglöf, Långsele, Sweden). Twig and trunk samples were fixed with 3% aqueous glutaraldehyde, which was adjusted from 25% aqueous glutaraldehyde (Nacalai Tesque, Inc., Kyoto, Japan) diluted with distilled water after sampling (approximately within one hour).

Twig and trunk samples and leaves were collected biweekly between 14 March and 4 July 2006; trunk samples were also collected monthly between 4 July and 29 August 2006; twig samples and leaves were also collected on 10 October and 7 November 2006, and 23 January 2007. We used the flush leaves, which flush immediately in spring [54], in the analysis of leaf area time courses.

### 4.3. Seasonal Changes in Leaf Area

The time courses of leaf area change were determined based on the methods previously described by Takahashi and Takahashi [30], with a slight modification. From scanned images of 3–10 leaf samples, leaf area was measured using Adobe Photoshop CC (Adobe, San Jose, CA, USA). The time courses of leaf area were calculated from the means of the sample leaf area. A nonlinear regression model of the leaf area time courses was fitted using Richards’ growth function [55,56] and the Solver function of Microsoft Excel: Richards’ function is

W = A (1 − b × e^−k(t−t0)^)^1/(1−m)^, (1)

where W is the leaf size (cm^2^) at time t (day of year (DOY), days); b, k, m are constants (b = 1, k = 0.00–0.50, m = 0.0–2.0); and t_0_ is time t at W = 0. The ultimate limiting value A (cm^2^) was defined as the maximum leaf area, and full leaf expansion was conveniently defined as the leaf area reaching 95% of value A. The duration of leaf area increase between 0% and 95% of value A (0%LA and 95%LA, respectively) was compared with the time of vessel formation in twigs and trunks.

### 4.4. Assessment of Vessel Formation

Transverse sections of twig or trunk samples were cut 15–30 μm thickness using a sliding microtome (TU-213, Yamato Kohki Industrial co., Ltd., Saitama, Japan). The sections were double-stained using safranin (1% in 50% ethanol; Nacalai Tesque, Inc., Kyoto, Japan) and fast green (1% in 95% ethanol; Nacalai Tesque, inc., Kyoto, Japan), dehydrated using a series of solutions with increasing ethanol (50%, 70%, 80%, 90%, 95%, and 100%), followed by replacement of ethanol with xylene (Nacalai Tesque, Inc., Kyoto, Japan), before mounting in Canada balsam (Nacalai Tesque, Inc., Kyoto, Japan) on glass slides [57], for observation with a light microscope (BX-50-32, Olympus co., Tokyo, Japan).

Vessels were lignified after cell wall deposition and before the disintegration of end walls [58,59,60,61]. Lignification of the first-formed vessels, which were often formed adjacent to the annual ring border in the current growth ring, was determined by the red color of safranin staining [57,58,62]. The phloroglucinol–hydrochloric acid reaction was used when double-staining yielded ambiguous results regarding lignification [62]. In the present study, first-formed vessel formation in twigs and trunks was divided into four classes: no vessel (there was no first-formed vessel in the present year), vessel enlargement (the first-formed vessels were enlarged but their walls were not lignified), at least one of the first-formed vessels lignified, and almost all of the first-formed vessels lignified. Trunk samples were not collected after 20 June until 1 or 29 August in sample trees of Ls-183, Qg-174, and Qmy-146. When the first-formed vessels of trunks were enlarged but their walls were not lignified by 20 June or 4 July, and the first-formed vessels were lignified on 1 or 29 August, the date of the lignification was judged to be 2 weeks later than 20 June or 4 July in sample trees of Cca-171 and -172; Sp-151, -154, and -155; and Qg-160. When the first-formed vessels of trunks were enlarged by 1 August, the first-formed vessel lignification date was assessed to be 2 weeks later than 1 August in a sample tree of Qmy-148.

Deviation of the DOY of almost all of the first-formed vessel lignification in twigs and trunks from the DOY of 95%LA was calculated. The deviations were compared among tree species.

## 5. Conclusions

In this study, we found that the timing of first-formed vessel formation of twigs on the basis of full leaf expansion is the same among all species. We clarified that a relationship exists between vessel porosity and the timing of the first-formed vessel formation of trunks on the basis of full leaf expansion in ring-porous evergreen/deciduous species and diffuse-porous deciduous species. Ring-porous species, regardless of whether they are evergreen or deciduous, undergo leaf expansion and lignification of the first-formed vessels of the trunk at the same time, whereas these processes occur at different times in diffuse-porous deciduous species and radial-porous evergreen species. Some sample trees of diffuse-porous evergreen species and evergreen ring-(radial-)porous species had lignified first-formed vessels prior to full leaf expansion, though these species may transport water using several outer rings. Semi-ring-porous deciduous *A. sciadophylloides* also showed traits of the first-formed vessel lignification of trunks both before and after full leaf expansion. These differences in the timing of leaf/vessel formation among tree species are a reflection of the diversity in their life strategies. Further examinations are necessary to clarify the internal and external factors affecting the timing of trunk vessel formation and to evaluate the environmental conditions advantageous for the growth and life of trees that simultaneously form leaves and vessels.

## Figures and Tables

**Table 1:** Description of sample trees.

Leaf Habit.	Vessel Arrangement ^1^	Species ^2^	Family	DBH ^3^ (cm)	Tree Height (m)	Number of Trees
Deciduous	Ring-porous	*Quercus serrata* Murray	Fagaceae	23–36	8–14	3
	Diffuse-porous	*Liquidambar styraciflua* L.	Hamamelidaceae	22–56	22–29	4
	Semi-ring-porous	*Acanthopanax sciadophylloides* Franch. et Sav.	Araliaceae	16–29	7–17	5
Evergreen	Ring-(radial-)porous	*Castanopsis cuspidata* (Thunb.) Schottky	Fagaceae	12–56	7–26	3
	Diffuse-porous	*Cinnamomum camphora* (L.) J.Presl	Lauraceae	29–45	15–19	3
		*Ilex pedunculosa* Miq.	Aquifoliaceae	19–24	8–11	4
		*Symplocos prunifolia* Siebold et Zucc.	Symplocaceae	19–24	7–9	5
	Radial-porous	*Quercus glauca* Thunb.	Fagaceae	24–44	11–17	4
		*Quercus myrsinifolia* Blume	Fagaceae	22–53	15–20	4

^1^ Classes of porosity were defined based on Wheeler et al. [1], Hayashi [48], Itoh [49,50], the FFPRI website [51], and the InsideWood website [52]. Radial-porous species were defined by Gasson [2] and Noshiro and Sasaki [3]. ^2^ Scientific names are based on Yonekura and Kajita [53]. ^3^ Diameter at breast height = 1.3 ± 0.3 m above the ground.

## Data Availability

The data presented in this study are available in the article.
